# [(WR)_8_WKβA]-Doxorubicin Conjugate: A Delivery System to Overcome Multi-Drug Resistance against Doxorubicin

**DOI:** 10.3390/cells11020301

**Published:** 2022-01-16

**Authors:** Khalid Zoghebi, Hamidreza Montazeri Aliabadi, Rakesh Kumar Tiwari, Keykavous Parang

**Affiliations:** 1 Center for Targeted Drug Delivery, Department of Biomedical and Pharmaceutical Sciences, Harry and Diane Rinker Health Science Campus, Chapman University School of Pharmacy, Irvine, CA 92618, USA; zoghebi@chapman.edu (K.Z.); montazer@chapman.edu (H.M.A.); 2 Department of Pharmaceutical Chemistry, College of Pharmacy, Jazan University, Jazan 82826, Saudi Arabia

**Keywords:** antiproliferative, cancer, Doxorubicin, endocytosis, resistance

## Abstract

Doxorubicin (Dox) is an anthracycline chemotherapeutic agent used to treat breast, leukemia, and lymphoma malignancies. However, cardiotoxicity and inherent acquired resistance are major drawbacks, limiting its clinical application. We have previously shown that cyclic peptide [WR]_9_ containing alternate tryptophan (W) and arginine (R) residues acts as an efficient molecular transporter. An amphiphilic cyclic peptide containing a lysine (K) residue and alternative W and R was conjugated through a free side chain amino group with Dox via a glutarate linker to afford [(WR)_8_WKβA]-Dox conjugate. Antiproliferative assays were performed in different cancer cell lines using the conjugate and the corresponding physical mixture of the peptide and Dox to evaluate the effectiveness of synthesized conjugate compared to the parent drug alone. [(WR)_8_WKβA]-Dox conjugate showed higher antiproliferative activity at 10 µM and 5 µM than Dox alone at 5 μM. The conjugate inhibited the cell viability of ovarian adenocarcinoma (SK-OV-3) by 59% and the triple-negative breast cancer cells MDA-MB-231 and MCF-7 by 71% and 77%, respectively, at a concentration of 5 μM after 72 h of incubation. In contrast, Dox inhibited the proliferation of SK-OV-3, MDA-MB-231, and MCF-7 by 35%, 63%, and 57%, respectively. Furthermore, [(WR)_8_WKβA]-Dox conjugate (5 µM) inhibited the cell viability of Dox-resistant cells (MES-SA/MX2) by 92%, while the viability of cells incubated with free Dox was only 15% at 5 μM. Confocal microscopy images confirmed the ability of both Dox conjugate and the physical mixture of the peptide with the drug to deliver Dox through an endocytosis-independent pathway, as the uptake was not inhibited in the presence of endocytosis inhibitors. The stability of Dox conjugate was observed at different time intervals using analytical HPLC when the conjugate was incubated with 25% human serum. Half-life (t_1/2_) for [(WR)_8_WKβA]-Dox conjugate was (∼6 h), and more than 80% of the conjugate was degraded at 12 h. The release of free Dox was assessed intracellularly using the CCRF-CEM cell line. The experiment demonstrated that approximately 100% of free Dox was released from the conjugate intracellularly within 72 h. These data confirm the ability of the cyclic cell-penetrating peptide containing tryptophan and arginine residues as an efficient tool for delivery of Dox and for overcoming resistance to it.

## 1. Introduction

Doxorubicin (Dox) is an anthracycline chemotherapeutic agent. Generally, Dox inhibits the synthesis of DNA by blocking the replication and transcription processes via intercalation with the base pairs of DNA double helix. Furthermore, Dox inhibits topoisomerase II (Top2β), which is an enzyme regulating DNA cut/reseal, thus preventing DNA replication, transcription, and repair. Dox activity includes the generation of free radicals that induce oxidative stress damage, resulting in cleavage or degradation of DNA [1].

Dox is one of the most effective chemotherapeutics and is indicated by the Food and Drug Administration (FDA) for a number of cancer conditions such as leukemia, breast cancer, ovarian cancer, neuroblastoma, soft tissue and bone sarcoma, bladder carcinoma, Hodgkin’s disease, malignant lymphoma, thyroid cancer, gastric carcinoma, and bronchogenic carcinoma [2]. 

As a defense mechanism, cancer cells often develop resistance after initial exposure to several chemotherapeutic drugs. One of the major drawbacks limiting Dox clinical application is inherent acquired resistance developed by different tumor cells. Resistance to chemotherapeutics is known as multidrug resistance (MDR), and includes different mechanisms such as enhanced drug efflux out of the cells via ATP-binding cassette family (ABC) transporters, alteration of intracellular target molecules, activation of DNA repair enzymes, and modulation of apoptotic pathways [3,4]. Among these, the overexpression of ATP-dependent efflux pump membrane proteins such as permeability glycoprotein (P-gp) is the most frequent [5]. Intracellular Dox accumulation is dependent on multiple factors, including cellular uptake, nuclear localization, cellular retention, and low efflux from the cells [6]. (P-gp) overexpression efficiently removes Dox and reduces its intracellular concentration [5]. 

Moreover, Dox exhibits undesirable pharmacokinetic properties such as low bioavailability, high volume of distribution, rapid blood excretion, and short half-life due to its hydrophilicity [7]. Consequently, extremely high doses are required in cancer chemotherapy to elicit therapeutic effects, leading to the possibility of many side effects, including the most severe, congestive heart failure due to cardiotoxicity [8]. 

Chemical conjugation with a parent drug has been a widely used drug delivery system, and is referred to as a prodrug strategy [9,10]. Anticancer drugs’ biological activity and adverse events can be altered using drug delivery systems, modifying physicochemical properties such as lipophilicity and enhanced cellular uptake, and increasing the activity through chemical conjugation with different chemical moieties. Many techniques have been developed to improve Dox delivery, such as using liposomes [11], gold nanoparticles [12], peptides [13,14,15], metal nanoparticles [16], and other covalent/noncovalent systems [17]. Drug delivery systems can be used to overcome multidrug resistance proteins (MRPs) efflux pumps involved in Dox resistance by different cancer types. 

Conjugation of Dox with cell-penetrating peptides (CPPs) has been employed as one of the most effective methods to translocate the drug into various cancer cell lines. Different linear CPPs such as penetratin [15], TAT [18], polyarginine [19], and maurocalcine [20] have been conjugated with Dox. These conjugates were found to be active against Dox-resistant cancer cell lines. For example, Dox-D-Penetratin showed potent activity against resistance K562/ADR (human erythroleukemic cells) as compared with Dox alone. The 50% inhibitory concentration (IC_50_) for Dox in K562/ADR was 65 ± 7 μM, whereas the IC_50_ of the conjugate in the same cells was 3 ± 1.4 μM [21]. In addition, Dox–TAT conjugate was tested on drug-resistant MCF-7 cells (MCF-7/ADR), and it was found that the conjugate was 8–10 times more potent than that of free Dox [22]. However, the internalization mechanism of those peptide-based delivery systems involves the endocytosis pathway. A number of cyclic CPPs as noncovalent nuclear targeting molecular transporters of Dox has previously been reported [23]. 

Cyclic peptides containing alternative arginine (R) and tryptophan (W) residues [WR]_n_ (*n* = 4–5) were found to be appropriate noncovalent carriers for Dox. We have previously reported the conjugation of cyclic CPPs such as [WR]_5_ with enhanced intracellular delivery of camptothecin (CPT), paclitaxel (PTX), Dox, and curcumin in different cancer cell lines with endosomal escape [24,25,26]. Cyclic [W(RW)_4_]-Dox enhanced the antiproliferative activity of Dox compared to the corresponding linear (RW)_4_–Dox in human leukemia (CCRF-CEM), ovarian adenocarcinoma (SK-OV-3), colorectal carcinoma (HCT-116), and breast carcinoma (MDA-MB-468). [W(RW)_4_]−Dox significantly improved the parent drug’s cellular uptake and retention time in SK-OV-3 cancer cells. Flow cytometry analysis exhibited 3.3–3.6-fold higher cellular uptake of cyclic conjugate than parent Dox alone and the corresponding physical mixtures, linear (RW)_4_ + Dox, in SK-OV-3 and cyclic [W(RW)_4_] + Dox cells after 24 h incubation [23]. 

Recently, we have reported amphiphilic cyclic CPPs composed of increasing numbers of W and R residues [WR]_6–9_, and compared their efficiency with the previously published cyclic [WR]_5_ in improving the cellular uptake of cell-impermeable compounds and their molecular transporter efficiency [27]. Among all synthesized peptides, [WR]_9_ was found to be the most effective peptide as a molecular transporter of fluorescence-labeled phosphopeptide (F′-GpYEEI) by 20-fold, compared to 4-fold when [WR]_5_ was used. Furthermore, [WR]_9_ has shown potent protein kinase (a protein that has been shown to be associated with unregulated cell signal transduction in cancer cells) inhibitory activity when compared with other synthesized peptides [WR]_5–8_ [28]. Herein, we report the preparation of an amphiphilic cyclic peptide containing alternative tryptophan (W) and arginine (R) residues and a lysine containing a free side chain amino group through the covalent conjugation with a 3-carbon chain linker attached to Dox 14-hydroxyl group to afford [(WR)_8_WKβA]-Dox conjugate. The peptide-Dox conjugate was designed to improve cellular uptake, prolong biological activity, and overcome intrinsic cellular efflux of the parent drug. The antiproliferative activity of the peptide-Dox conjugate was evaluated in multiple cancer and Dox-resistant cell lines. The cellular and mechanism of uptake were investigated in the presence of endocytosis inhibitors. Cellular hydrolysis and release of free Dox were evaluated in the human leukemia (CCRF-CEM) cell line.

## 2. Materials and Methods

All protected amino acids and resins were purchased from AAPPTEC (Louisville, KY, USA). Dox was purchased from LC Laboratories (Woburn, MA, USA). All the other chemicals reagents were purchased from MilliporeSigma (Milwaukee, WI, USA). Medium (RPMI-1640), fetal bovine serum, and all other cell biology reagents were purchased from Wilken Scientific (Pawtucket, RI, USA) and Fisher Scientific (Hanover Park, IL, USA). The final products were characterized by high-resolution matrix-assisted laser desorption/ionization time-of-flight (MALDI-TOF, GT 0264) from Bruker Inc. (Billerica, MA, USA) with α-cyano-4-hydroxycinnamic acid as a matrix. The final crude products were purified by a reversed-phase high-performance liquid chromatography (RP-HPLC) from Shimadzu (LC-20AP) (Canby, OR, USA) by using a gradient system of water and acetonitrile and a reversed-phase preparative column C18 (XBridge BEH130 from Waters) (Milford, MA, USA). The final peptide-Dox conjugate had a purity of >95%, as shown by spectroscopy and analytical HPLC data (Supporting Information, Figures S6 and S7). 

Human leukemia carcinoma cell line (CCRF-CEM, ATCC No. CCL-119), human ovarian adenocarcinoma cells (SK-OV-3, ATCC No. HTB-77) cells, human breast adenocarcinoma cells (MDA-MB-231, ATCC No. CRM-HTB-26 and MCF-7, ATCC No. HTB-22), uterine sarcoma cells (MES-SA/MX2, ATCC No. CRL-2274), and heart/myocardium cells (H9C2, ATCC No. CRL 1446) were obtained from American Type Culture Collection (ATCC, Manassas, VA, USA). VECTASHIELD VIBRANCE with DAPI (used to stain the cell nuclei) was obtained from Vector Laboratories (Burlingame, CA, USA). Cell Titer 96^®^ AQueous MTS Reagent was obtained from Promega (Madison, WI, USA). An MTS reagent composed of a tetrazolium derivative (3-(4,5-dimethylthiazol-2-yl)-5-(3-carboxymethoxyphenyl)-2-(4-sulfophenyl)-2H-tetrazolium (named MTS) and phenazine ethosulfate (PES) was used for the cell-based proliferation studies. All the materials for cell culture studies were purchased from Fischer Scientific (Hanover Park, IL, USA).

### 2.1. Chemical Synthesis

#### 2.1.1. Synthesis of Cyclic Peptide [(WR)_8_WKβA]

Fmoc solid-phase peptide synthesis followed by solution-phase cyclization was utilized to synthesize cyclic [(WR)_8_WKβA]. Fmoc-Arg(Pbf)-OH, Fmoc-Trp(Boc)-OH, Boc-βAla-OH, and Dde-Lys(Fmoc)-OH were used as building block amino acids in the peptide synthesis. Preloaded resin, H-Trp(Boc)-2-chlorotrityl resin (0.44 meq/g, 0.4 mmol, 905 mg) was swelled in *N*,*N*-dimethylformamide (DMF) under dry nitrogen gas (3 × 15 min). After filtration of the solvent, the next Fmoc-protected amino acid (3 equiv.) was conjugated to the free *N*-terminal in the presence of 2-(1H-benzotriazol-1-yl)-1,1,3,3-tetramethyluronium hexafluorophosphate (HBTU) (3 equiv.) as a coupling reagent and *N*,*N*-diisopropylethylamine (DIPEA) (6 equiv.) as a base in DMF by agitating under dry nitrogen gas for 1 h. After the completion of the coupling, the reaction solution was filtered off. The resin was washed extensively with DMF (15 mL, 2 × 5 min). Fmoc deprotection was performed in the presence of piperidine in DMF (10 mL, 2 × 15 min, 20% *v*/*v*). The reaction solution was filtered off and the resin was washed with DMF (15 mL, 2 × 5 min). The subsequent amino acids were coupled and deprotected as described above. After the final coupling with Boc-βAla-OH, the resin was washed with DMF (3 × 25 mL, each time 5 min). Then, the Dde group at the *N*-terminal of lysine was deprotected by using hydrazine monohydrate (2% *v*/*v*) solution in DMF (3 × 20 mL, each time 10 min), and the resin was washed with DMF (5 × 50 mL) followed by washing with DCM (3 × 50 mL). The side chain-protected peptide was cleaved from trityl resin using a cleavage cocktail containing dichloromethane, trifluoroethanol, acetic acid (DCM:TFE:AcOH, 7:2:1 *v*/*v*/*v*, 50 mL), shaking for 2.5 h at room temperature to yield the side chain-protected linear peptide. The resin was collected by filtration and washed with TFE/DCM (2:8 *v*/*v*, 2 × 10 mL). The combined filtrates were evaporated under reduced pressure. Hexane (2 × 25 mL) and DCM (1 × 25 mL) were added to the residue to remove the acetic acid from the cleaved crude peptide. The crude peptide was obtained as a white solid and was dried in a vacuum overnight. The compound was directly used for the next cyclization reaction. The linear peptide was dissolved in anhydrous DMF/DCM (5:1 *v*/*v*, 250 mL). 1-Hydroxy-7-azabenzotriazole (HOAt, 223 mg, 1.64 mmol, 4 equiv) and *N*,*N*-diisopropylcarbodiimide (DIC, 290 μL, 1.86 mmol, 4.5 equiv.) were added to the mixture, and the solution was stirred at room temperature overnight. The completion of the cyclization was confirmed by MALDI-TOF. The solvent was removed by using a rotary evaporator under low pressure. The crude cyclic peptide was dried overnight, and a cleavage cocktail composed of trifluoracetic acid (TFA), anisole, thioanisole (9:1:2 *v*/*v*/*v*), and DTT (50 mg, dithiothreitol) (20 mL total volume) was mixed with the crude product for 6 h to remove the protecting groups on the side chains. Cold diethyl ether was added to precipitate the crude peptide, which was then centrifuged and separated. The molecular weight of the cyclic peptide was confirmed again with MALDI-TOF (Figure S5, Supporting Information). The cyclic peptide was purified using RP-HPLC and lyophilized.

[(WR)_8_WKβA]: MALDI-TOF (m/z): C_156_H_203_N_53_O_19_, calculated: 3122.6548, found: 3122.6093 [M]^+^.

#### 2.1.2. Synthesis of N-Fmoc-Dox-14-O-Hemiglutarate

For the synthesis of *N*-Fmoc Dox derivative, Dox hydrochloride was dissolved in anhydrous DMF (5 mL) under a nitrogen atmosphere. Then Fmoc-OSu was slowly added to the reaction mixture, followed by dropwise addition of anhydrous *N*,*N*-diisopropylethylamine. After stirring the reaction overnight at room temperature and in dark conditions, the reaction was stopped and the solvent was removed. The oily liquid was triturated with TFA solution in water (0.1% *v*/*v*) to afford a solid crystal. The solid was collected after filtration and washed with cold diethyl ether to remove traces of excess of Fmoc-OSu. The pure Fmoc-*N*-Dox was reacted with glutaric anhydride in the presence of anhydrous DIPEA in anhydrous DMF for 16 h under nitrogen atmosphere. The final compound was purified by HPLC to afford pure *N*-Fmoc-Dox-14-*O*-hemiglutarate. 

#### 2.1.3. [(WR)_8_WKβA]-Dox Conjugate Synthesis

*N*-Fmoc-Dox-14-*O*-hemiglutarate (5 mg), cyclic peptide [(WR)_8_WKβA] (10 mg), benzotriazole-1-yloxytripyrrolidinophosphonium hexafluorophosphate (PyBOP, 30 mg), and 1-hydroxybenzotriazole (HOBt, 30 mg) were added to the glass vial under nitrogen atmosphere. The mixture was stirred to dissolve the compounds, followed by the addition of DIPEA (80 µL) in anhydrous DMF (5 mL). The mixture was stirred for 2 h in dark conditions. The solvent was then removed, and cold diethyl ether was added to the residue. The crude cyclic peptide was precipitated and centrifuged to obtain the crude solid peptide-Dox conjugate. A solution of piperidine in DMF was used (20% *v*/*v*, 2 mL for 5 min) to remove the Fmoc protecting group. The solution color turned blue, and the reaction was terminated by adding drops of TFA solution in DMF (20% *v*/*v*) until the solution color turned red. The solvent was removed under reduced pressure. The product was then dissolved in acetonitrile/water (50% *v*/*v*). The final product was purified using HPLC and characterized by using MALDI-TOF (Figure S6, Supporting Information).

[(WR)_8_WKβA]-Dox: MALDI-TOF (m/z): C_187_H_234_N_54_O_32_ calculated: 3747.8343, found: 3747.8257 [M]^+^, 3225.2231 [[(WR)_8_WKβA]-Glutarate + 3H]^+^.

### 2.2. In Vitro Cytotoxicity Assay of [(WR)_8_WKβA]-Dox Conjugate

Cytotoxic activity of the synthesized conjugate and noncovalent mixtures of cyclic [WR]_9_ + Dox was determined by using SK-OV-3, MDA-MB-231, MCF-7, MES-SA/MX2, and H9C2 cells, according to the previously reported procedure. In brief, the cells were seeded at 5000 cells (0.1 mL per well in 96-well plates). An appropriate growth medium was used for each cell line (for SK-OV-3: McCoy’s 5A with L-Glutamine containing fetal bovine serum (FBS) (10%) and penicillin or streptomycin (1%); for MDA-MB-231 and MCF-7 cells: DMEM/F12 (1:1) (1×) with L-Glutamine and 15 mM HEPES containing FBS (10%) and penicillin or streptomycin (1%), and for MES-SA/MX2 and H9C2 cells: Minimum Essential Medium Eagle with Earle’s salts and sodium bicarbonate, without L-glutamine containing FBS (10%), and penicillin or streptomycin (1%)). The cells were seeded in a complete growth medium 24 h prior to the experiment. The compounds at different concentrations (1–10 µM) were added to each well in triplicate and incubated for 24 and 72 h at 37 °C in a humidified atmosphere of 5% CO_2_. Dox (5 µM) was used as a positive control, while water and cell culture medium were used as negative controls. The compounds were dissolved in water; hence, the water was used as a negative control to normalize the data. After the incubation period, MTS reagent (20 µL) was added to each well. The incubation was continued for 3 h. The MTS protocol is based on the reduction reaction of MTS tetrazolium by the viable cells. Cell viability was then measured by the determination of the fluorescence intensity at 490 nm using a SpectraMax M2 microplate spectrophotometer. The percentage of cell viability was then calculated using the following equation:

[(OD value of cells treated with the compound) − (OD value of culture medium)]/[(OD value of control cells) − (OD value of culture medium)] × 100%.

### 2.3. Confocal Microscopy

MDA-MB-231, SK-OV-3, MES-SA/MX2, and H9C2 cells (7 × 10^4^ cells/well) were seeded with a medium on a coverslip 24 h prior to the experiment in six well plates. After 24 h, the medium was changed with opti-MEM. The cells were treated with Dox and peptide-Dox conjugate (5 μM) in opti-MEM for 24 h. After 24 h incubation, the media were removed, and cells were washed three times with PBS in each well. Then, the cells were fixed with 3.7% formaldehyde for 10 min, followed by washing three times with PBS for 5 min (pH 7.6). DAPI (40 µL) for staining the nuclei was placed on a microscope slide, and it was covered with the coverslip with the cell-attached side facing down. The slides were left standing horizontally in a dark place with airflow to allow drying faster. The cells were photographed using Nikon Instruments A1 Confocal Laser Microscope Series (Nikon Instruments Inc, NY, USA) and NIS-Elements software (AR 4.30.02, 64 bit). The scan mode was selected as Galvano. The magnification and resolution were set at 40× and 1024, respectively. 

### 2.4. Differential Extraction of Dox in Nuclear and Cytoplasmic Compartments

In order to confirm nuclear delivery of peptide-conjugated Dox, in addition to confocal microscopy, we performed a differential nuclear and cytoplasmic extraction. Cells were exposed to (50 μM) free and conjugated Dox for 4 h and incubated at 37 °C in a humidified atmosphere of 5% CO_2_. The cells were then pelleted at 2000 rpm for 5 min. After removing the supernatant, the cells were resuspended in Dounce buffer containing protease inhibitors. The cell suspension to be used was incubated on ice for 15 min before being spiked with cold 10% NP-40 and vortexed for 10 s. The suspension was centrifuged at 10,000 rpm for 10 min at 4 °C to pellet the nuclei. The supernatant was isolated for cytoplasmic extraction. The nuclear pellet was washed in cold PBS twice and centrifuged to remove the supernatant. The isolated nuclei were then lysed using RIPA buffer containing protease inhibitors. The nuclear and cytoplasmic extractions were analyzed by analytical HPLC to differentially quantify free Dox (or Dox released from the conjugates) and peptide-conjugate Dox using an Agilent Zorbax SB-C18 (4.6 × 150 mm) column. The column was equilibrated with 0.1% TFA-water (solvent A). The elution was performed at a flow rate of 1 mL/min using a gradient of acetonitrile from 0 to 35% in 50 min, with the absorbance measured at 490 nm.

### 2.5. Fluorescence-Activated Cell Sorter (FACS) Analysis of Cellular Uptake in the Presence of Endocytosis Inhibitors 

A flow cytometry study was conducted in the presence of endocytosis inhibitors such as nystatin, chlorpromazine, chloroquine, and methyl β-cyclodextrin to determine whether the cellular uptake for the peptide-Dox was endocytosis-dependent. MDA-MB-231 (5 × 10^5^ cells/well) were seeded in the medium 24 h prior to the experiment in six well plates, and after 24 h the medium was changed with opti-MEM. The cells were preincubated by various endocytosis inhibitors including nystatin (50 μg/mL), chloroquine (100 μM), chlorpromazine (30 μM), and methyl-β-cyclodextrin (2.5 mM) for 30 min at 37 °C in a humidified atmosphere of 5% CO_2_. The cells were then incubated with [(WR)_8_WKβA]-Dox conjugate (5 µM) in the presence of inhibitors for 3 h. After 3 h incubation, the media containing the compounds were removed. Next, the cells were digested with 0.05% trypsin/EDTA (0.53 mM) for 5 min to remove any artificial surface binding, centrifuged at 2500 RPM for 5 min with Fisher Scientific accuSpin Micro 17, and then collected as the precipitant. Then the cells were washed two times with PBS, resuspended in flow cytometry buffer, and transferred to the flow cytometry tubes through the 35 µm Strainer Mesh cap. Finally, the cells were analyzed by flow cytometry (FACSCalibur: Becton Dickinson, San Jose, CA, USA) using the Propidium Iodide channel and CellQuest software. The data presented were based on the mean fluorescence intensity for 10,000 cells collected. All assays were performed in triplicate.

### 2.6. Stability Studies

Human serum (250 μL) was diluted by adding sterile water (650 μL) and conjugate stock solution (100 μL) to give a sample with 25% human serum and 5 μM conjugate. The sample was kept at 37 °C in an incubator to mimic human body temperature. At regular time intervals, aliquots (100 μL) were taken and diluted with methanol (200 μL). After centrifugation (7000 RPM) of the mixture for 10 min, the supernatant was analyzed by analytical HPLC at 495 nm, and the major HPLC peaks were analyzed by mass spectrometry. The mobile phase used was acetonitrile/water with 0.1% TFA, with a gradient of 20–45% and a flow rate of 1 mL/min in 50 min. The percentage of intact conjugate and the release of the free drug were plotted against incubation time. The AUC obtained after each run was used to confirm the conjugate’s degradation, and t_1/2_ (the time needed to hydrolyze half of the initial conjugate in human serum) was calculated from the graph plotted as AUC versus incubation time. 

### 2.7. Intracellular Hydrolysis

Intracellular hydrolysis of [(WR)_8_WKβA]-Dox and accumulation of free Dox and the peptide–Dox conjugate were determined in CCRF-CEM cells by HPLC analysis. Cells were grown with serum-free RPMI medium in 75 cm^2^ culture flasks to ∼70–80% confluence (1.37 × 10^7^ cells/mL). The medium was first replaced with fresh RPMI medium having [(WR)_8_WKβA]-Dox conjugate (5 μM). Then the cells were incubated for 4 h at 37 °C. After 4 h, the medium containing the conjugate was aspirated, and cells were washed three times with PBS before adding a fresh serum-free medium. The cells were first partitioned/transferred to culture plates (six wells) contained 1.37 × 10^7^ cells per well in 5 mL of medium, then incubated for the indicated time. After incubation, the cells were collected using centrifugation. After centrifugation, the medium was removed by decantation. The cell pellets were washed with ice-cold PBS to remove any remaining medium. The cell pellets were then completely extracted with an equal volume of methanol, chloroform, and isopropanol mixture (4:3:1 *v*/*v*/*v*) and finally filtered through 0.2 μm filters. The relative amount of Dox and [(WR)_8_WKβA]-Dox concentrations in cell lysates were quantified by analytical HPLC using the water/acetonitrile solvent. 

### 2.8. Data Analysis

The data are presented as the mean standard deviation for the stated number of samples. A significant difference test was performed using student’s *t*-test between two groups. For data over three groups, one-way ANOVA and post hoc Tukey tests were performed. The alpha threshold was set to 0.05 with a 95% confidence interval.

## 3. Results and Discussion

### 3.1. Chemistry

**Scheme 1:**
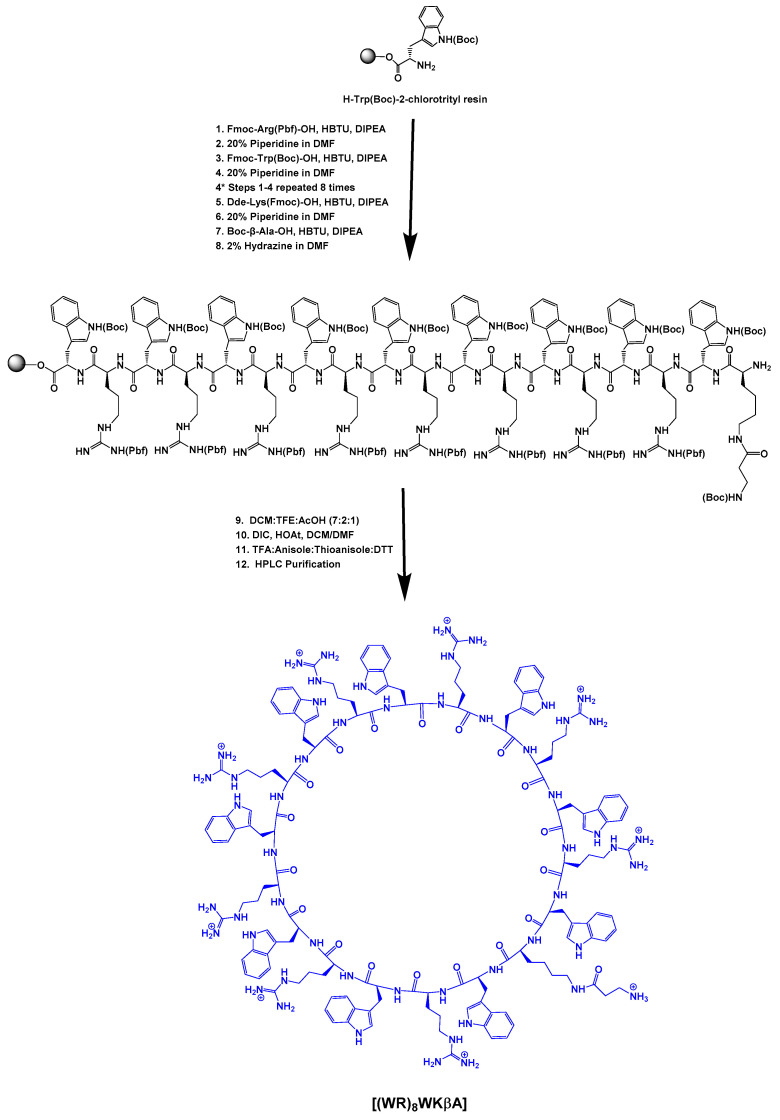
Synthesis of cyclic peptide [(WR)_8_WKβA].

Fmoc solid-phase peptide synthesis followed by solution-phase cyclization was utilized to synthesize cyclic [(WR)_8_WKβA]. The linear protected peptide (Dde-K(Boc-βA)(W(Boc)_9_R(Pbf)_8_) was assembled on the H-Trp(Boc)-2-chlorotrityl resin. The Dde group of *N*-terminal lysine was then deprotected in the presence of hydrazine (2% in DMF). The side chain-protected peptide was cleaved from the resin using AcOH/TFE/DCM (1:2:7 *v*/*v*/*v*) cocktail. The cyclization of the side chain-protected peptide was performed under pseudo-dilute conditions in the presence of HOAt and DIC (Scheme 1). The cyclic peptide was cleaved in the presence of reagent R, purified using reversed-phase HPLC, and used for conjugation with Dox. 

**Scheme 2:**
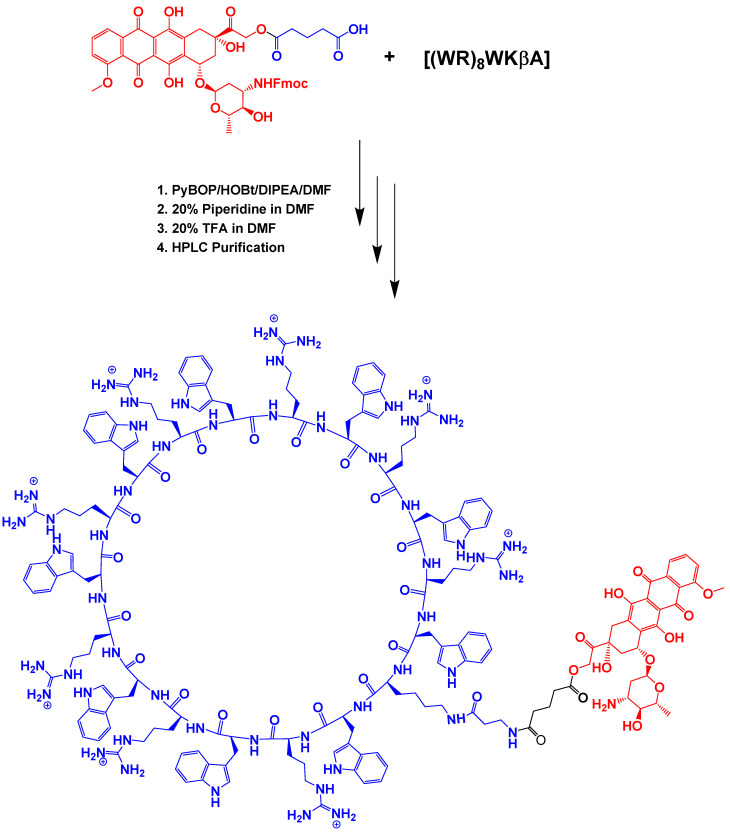
Synthesis of cyclic peptide [(WR)_8_WKβA]-Dox conjugate.

*N*-Fmoc-Dox-14-*O*-hemiglutarate was prepared as described previously. In brief, the reaction of Fmoc-protected Dox with glutaric anhydride was carried out to produce the Fmoc-Dox hemiglutarate ester with a free COOH, which after HPLC purification and lyophilization was used for coupling with fully deprotected cyclic peptide [(WR)_8_WKβA]. The conjugation of the cyclic peptide with *N*-Fmoc-Dox-14-*O*-hemiglutarate was achieved by a similar pattern. The equimolar amounts of the peptide and Dox were coupled through the reaction of the free amino group of [(WR)_8_WKβA] and carboxylic acid in the Fmoc-Dox hemiglutarate ester. The carboxylic group in Fmoc-protected Dox was pre-activated in the presence of HOBt/PyBOP/DIPEA in DMF for 15 min before reaction with the peptides. After conjugation, the Fmoc protecting group of Dox was removed using piperidine and was then acidified to yield [(WR)_8_WKβA]-Dox conjugate (Scheme 2) that was purified using HPLC and lyophilized. The structures of all the final compounds were confirmed using a high-resolution MALDI-TOF/TOF mass spectrometer (Figure S6, Supporting Information). The purity of the final product (≤95%) was confirmed by reversed-phase analytical HPLC using a gradient system with water (0.1% TFA) and acetonitrile as eluting solvents (Figure S7, Supporting Information). 

### 3.2. Biological Activities

#### 3.2.1. Antiproliferative Activity of [(WR)_8_WKβA]-Dox Conjugate 

The cytotoxicity of the synthesized conjugate was evaluated in SK-OV-3, triple-negative breast cancer (TNBC) wild-type MDA-MB-231 and MCF-7 cells, and multidrug resistance (MDR) MES-SA/MX2 cells to determine the effect of the peptide conjugation on antiproliferative efficacy in sensitive and resistant cell lines. The activity of synthesized conjugate (1, 5, and 10 μM) was evaluated in a comparative study with the noncovalent physical mixture of [WR]_9_ (1, 5, and 10 μM) + Dox (5 μM) and Dox alone (5 μM). We have previously reported the synthesis and evaluation of hybrid cyclic linear [R_5_K]W_7_A-Dox conjugate in different cancer cell lines [29]. The rationale for designing [(WR)_8_WKβA]-Dox conjugate was based on the fact that we found cyclic peptide [WR]_9_ composed of alternate R and W was a more potent kinase inhibitor than [WR]_5_ and [R_5_K]W_7_ against c-Src, Abl, PKCa, Braf, Cdk2/cyclin A1, and that Lck [28]. [WR]_9_ was a superior molecular transporter versus [WR]_5_ [27]. We hypothesized that a Dox conjugate of [(WR)_8_WKβA], a derivative of [WR]_9_, with a large cyclic ring of alternate R and W residues would have a higher antiproliferative activity than [R_5_K]W_7_A-Dox [29] and [W(RW)_4_]-Dox [23], as Dox conjugates of [R_5_K]W_7_ and [WR]_5_, respectively.

**Figure 1:**
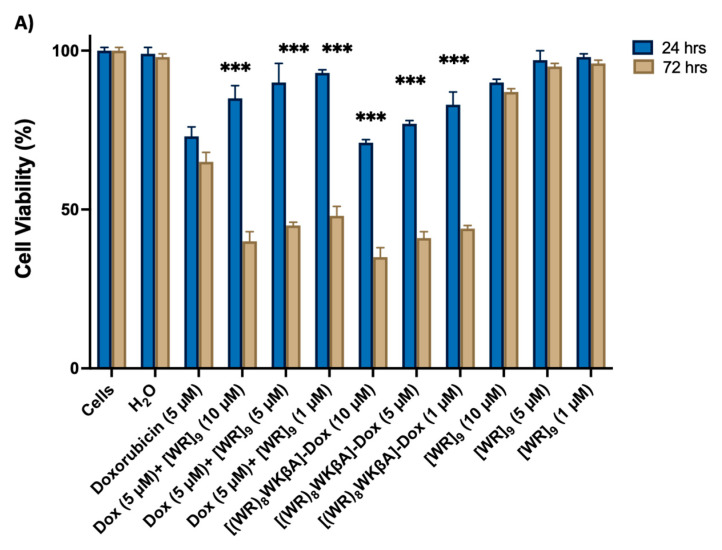

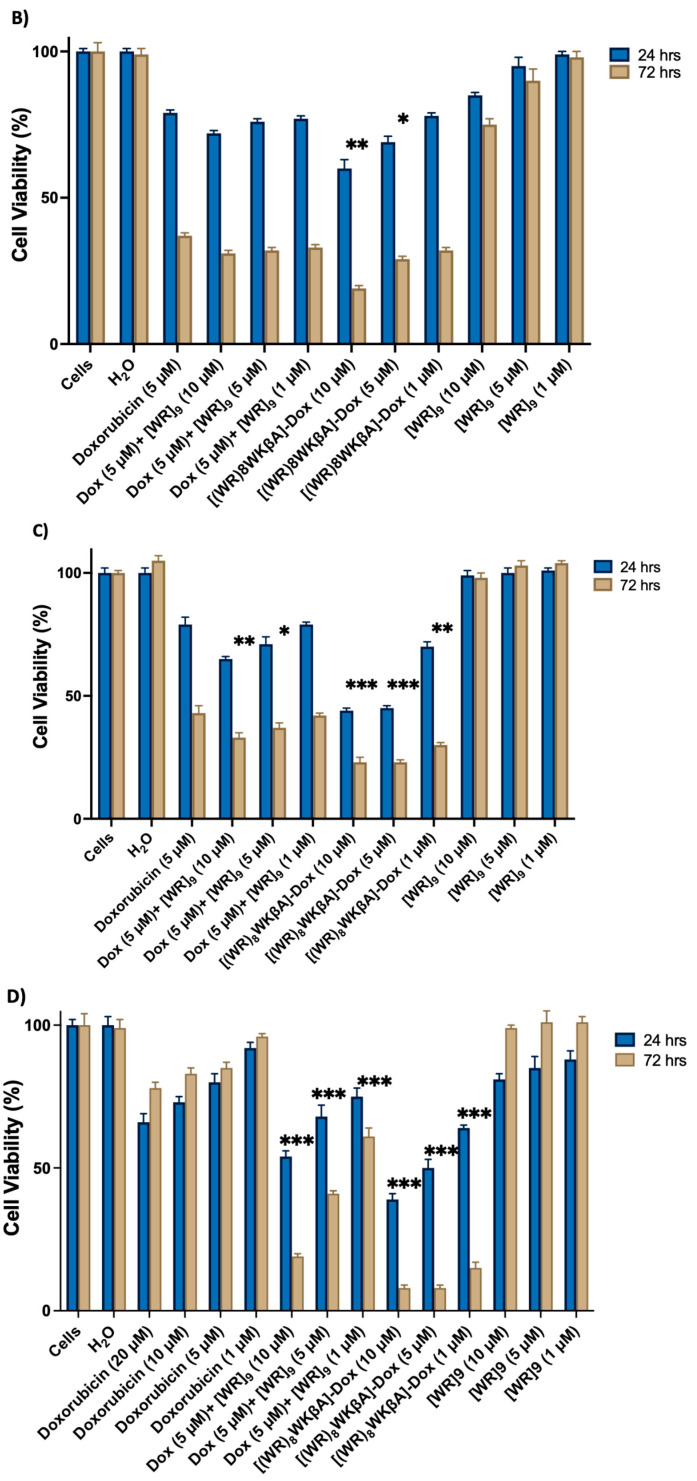
Inhibition of (**A**) SK-OV-3, (**B**) MDA-MB-231, (**C**) MCF-7, and (**D**) MES-SA/MX2 cells (Dox-resistant cells) by free Dox (5 µM) or (1, 5, 10, and 20 µM), [(WR)_8_WKbA]-Dox, [WR]_9_ + Dox, and [WR]_9_ at 1, 5, and 10 mM. Results are mean ± SD (*n* = 3) (* *p* < 0.05, ** *p* < 0.01, *** *p* < 0.001 treatment vs. Ctrl (Dox), *p*-value only for treatment after 72 h).

SK-OV-3 cells were exposed to [(WR)_8_WKβA]-Dox, [WR]_9_ + Dox at 1, 5, and 10 mM and Dox alone at 5 mM, as shown in Figure 1A. In general, the antiproliferative activities of [(WR)_8_WKβA]-Dox and the physical mixture [WR]_9_ + Dox were found to be time-dependent. When compared to free Dox, all treatment groups showed low antiproliferative activity after 24 h. However, the cell proliferation inhibitory activity of compounds was enhanced at a longer period of incubation (72 h) of compounds with SK-OV-3 cells, presumably due to the time-dependent hydrolysis of the [(WR)_8_WKβA]-Dox conjugate or time needed for the encapsulated Dox in the physical mixture [WR]_9_ + Dox to release free Dox. [(WR)_8_WKβA]-Dox conjugate was able to reduce the SK-OV-3 cell viability by 65%, 59%, and 56% at 10, 5, and 1 µM, respectively, after 72 h compared to Dox, which reduced the viability by 35% at 5 µM after 72 h incubation. Previously in this cell line at 72 h incubation, the cyclic [W(RW)_4_]−Dox inhibited the cell proliferation by 51% at a concentration of 1 μM [23], whereas [R_5_K]W_7_A-Dox was able to reduce the cell viability by 29, 39, and 48% at 5, 10, and 25 μM, respectively [29]. A similar pattern was observed when the physical mixture of [WR]_9_ and Dox was used. [WR]_9_ showed higher antiproliferative at 10, 5, and 1 µM when used with Dox at 5 µM, showing 40%, 45%, and 48% viability, respectively, when compared with free Dox alone after 72 h incubation. The peptide alone exhibited minimal cytotoxicity (90–98% viability) after 24 h at 10, 5, and 1 µM and (87–96% viability) at 72 h. 

The main option for the treatment of TNBC is chemotherapy [30]. However, its efficacy has been seriously compromised due to the development of multidrug resistance (MDR) [31]. Therefore, to test the therapeutic efficacy of [(WR)_8_WKβA]-Dox conjugate, cell cytotoxicity was evaluated using two TNBC cell lines (MDA-MB-231 and MCF-7). 

Cell viability was studied in MDA-MB-231 cells with different concentrations of [(WR)_8_WKβA]-Dox and [WR]_9_ (1, 5, and 10 μM) + Dox (5 μM), and Dox alone at 5 μM, as shown in Figure 1B. After 24 h, [(WR)_8_WKβA]-Dox showed lower cell viability of 60 and 69% at 10 and 5 µM, respectively, when compared with the conjugate showing cell viability of 79% at 1 µM. Dox alone showed 79% viability at 5 µM. The physical mixture of [WR]_9_ + Dox did not significantly reduce the viability of MDA-MB-231 cells compared to Dox after 24 h incubation. However, the antiproliferative activity of all compounds was enhanced at the 72 h incubation period. [(WR)_8_WKβA]-Dox conjugate showed significantly high antiproliferative activity against MDA-MB-231 cells, with only 19% and 29% cell viability at 10 and 5 µM, respectively. Interestingly, [(WR)_8_WKβA]-Dox conjugate showed comparable antiproliferative activity at 1 µM with 32% viability compared to control free Dox alone, which generated 37% viability. With 31–33% viability, the physical [WR]_9_+ Dox mixture did not significantly reduce cell viability compared to control Dox alone after 72 h incubation. [WR]_9_ alone showed minimal cytotoxicity (85–98% viability) after 24 h at 10, 5, and 1 µM and (75–98% viability) at 72 h. These data indicate that [(WR)_8_WKβA]-Dox conjugate has significantly higher antiproliferative activity against TNBC MDA-MB-231 than either Dox alone or the corresponding physical mixture when compared at similar concentrations and incubation times. 

Figure 1C exhibits the cell viability study in MCF-7 cells with [(WR)_8_WKβA]-Dox (1, 5, and 10 μM), physical mixture [WR]_9_ (1, 5, and 10 μM) + Dox (5 μM), and Dox alone at 5 μM. The conjugate showed significant antiproliferation activity when incubated with cells for 24 and 72 h at 10 and 5 µM. After 24 h, the conjugate reduced the cell proliferation by 56%, 55%, and 28% at 10, 5, and 1 µM, respectively, while Dox (5 µM) decreased proliferation by 21%. After 72 h, cell proliferation reduced by 77, 77, and 70% at 10, 5, and 1 µM, respectively, compared to 57% for Dox. A physical mixture of [WR]_9_ with Dox showed a significant reduction of 67% and 63% in cell viability after 72 h at 10 and 5 µM, respectively, while the physical mixture of [WR]_9_ (1 µM) with Dox (5 µM) demonstrated a comparable viability percentage to Dox alone. [WR]_9_ at different concentrations showed no significant effect on cell survival. These data further confirm that [(WR)_8_WKβA]-Dox is more effective against TNBC MCF-7 cells than Dox and the corresponding physical mixtures. 

The overexpression of ATP-dependent efflux pump membrane proteins such as P-gp is the most frequent mechanism reported for Dox resistance. P-gp overexpression efficiently removes Dox and reduces its intracellular concentration [32]. Thus, the efficacy of [(WR)_8_WKβA]-Dox conjugate in MDR cancer cells was investigated to explore the possibility of overcoming resistance to Dox as a result of overexpression of efflux membrane proteins. 

Uterine sarcoma cell line MES-SA/MX2 overexpresses P-gp and is a substrate for small molecules drugs such as Dox [33]. [(WR)_8_WKβA]-Dox conjugate and [WR]_9_ + Dox were significantly more effective than Dox alone (1, 5, 10, or 20 µM) in Dox-resistant MES-SA/MX2 cells. As expected, free Dox at 1, 5, 10, or 20 µM did not show significant efficiency against MES-SA/MX2, as shown in Figure 1D, while the conjugate was significantly more potent. At 24 h, the conjugate showed a significant reduction in cell viability by 39, 50, and 64% at 10, 5, and 1 µM, respectively, as shown in Figure 1D. The antiproliferative activity for [(WR)_8_WKβA]-Dox conjugate was significantly increased after 72 h, with only 8, 8, and 15% Dox-resistant cell survival at 10, 5, and 1 µM, respectively, compared to 85% for control Dox alone. Previously, it was found that [R_5_K]W_7_A-Dox conjugate exhibited minimal cytotoxicity (0–10%) after 72 h at 5 and 10 μM, whereas it was found to significantly reduce the cell viability by almost 80% at 25 μM [29]. In addition, [WR]_9_ + Dox mixture demonstrated significantly higher antiproliferative activity than Dox at 24 and 72 h. However, the activities of the physical mixtures were less than [(WR)_8_WKβA]-Dox conjugate. The maximum antiproliferative activity for the physical mixture was observed at a 1:2 ratio (5 µM:10 µM) of Dox:[WR]_9_, which showed 46 and 71% reduction in cell survival after 24 and 72 h, respectively. The physical mixture in 1:1 ratio (5 µM:5 µM) of Dox:[WR]_9_ showed significantly diminished cell viability, with 32 and 60% after 24 and 72 h, respectively, while [WR]_9_ alone had no significant antiproliferative effect after 24 and 72 h. 

Cardiotoxicity associated with Dox therapy is well-documented. To investigate the effect of peptide conjugation on Dox cardiotoxicity, rat H9C2 myocardium cells were exposed to [(WR)_8_WKβA]-Dox conjugate, Dox, and the physical mixture of [WR]_9_ with Dox for 24 h. The results indicated that no significant cytotoxicity was observed for [(WR)_8_WKβA]-Dox conjugate at concentrations of 1 and 5 µM, while at 10 µM, the cytotoxicity of [(WR)_8_WKβA]-Dox conjugate was comparable to Dox and the physical mixture of Dox (5 µM) with [WR]_9_ (10 µM) (Figure S1, Supporting Information). Confocal microscopy also confirmed high uptake of Dox (5 µM) and physical mixture of Dox (5 µM) with [WR]_9_ (5 µM) or [WR]_9_ (10 µM) in heart cells, consistent with the cytotoxicity data, while [(WR)_8_WKβA]-Dox conjugate at 5 µM did not show any significant uptake (Figure S2, Supporting Information). 

#### 3.2.2. Cellular Internalization

**Figure 2:**
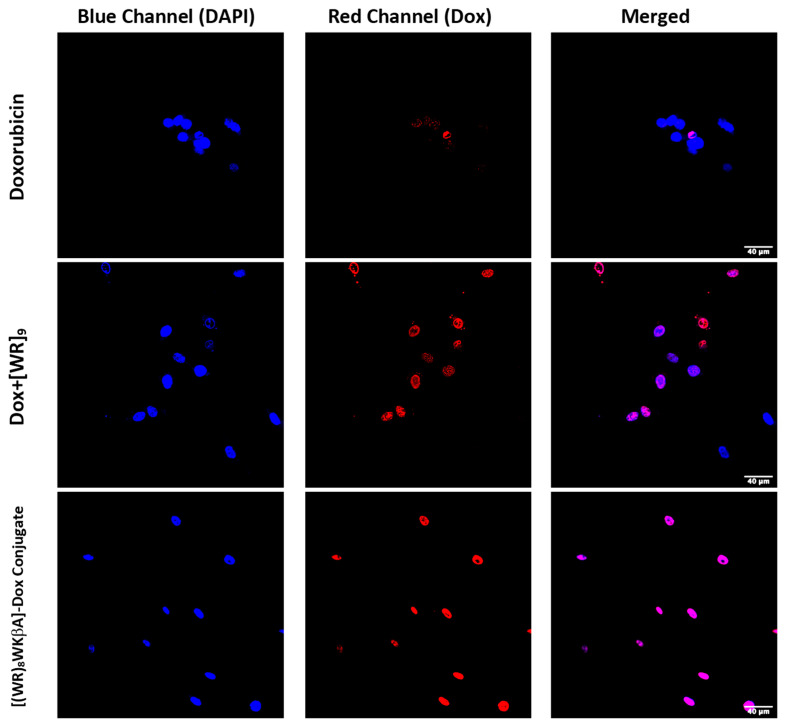
Confocal microscopy images of free Dox (5 μM), [WR]_9_ + Dox (1:1, (5 μM)) [(WR)_8_WKβA]-Dox conjugate (5 μM), or after 24 h in SK-OV-3 cells. Red represents the fluorescence of Dox.

Confocal microscopy was used to evaluate, visualize and confirm the internalization of [(WR)_8_WKβA]-Dox conjugate vs. free Dox (Figure 2). The cellular internalization was evaluated in Dox-sensitive/resistant cells to study the effect of overexpression of the MDR efflux proteins on the Dox internalization by the peptide conjugate. Cells were treated with Dox, [(WR)_8_WKβA]-Dox conjugate, and the physical mixture [WR]_9_ + Dox (1:1) for 24 h, and the nuclei were stained with DAPI. The cellular internalization of all test compounds was examined first in SK-OV-3 cells (Figure 2). To achieve the primary influx, the cells were incubated with Dox (5 µM), [(WR)_8_WKβA]-Dox conjugate (5 μM), and [WR]_9_ + Dox (1:1, (5 μM)) for 3 h. This process was followed by 24 h incubation with media to allow the cells to start the efflux process through pumping the compounds out. SK-OV-3 cells have an efflux mechanism for Dox after 24 h that leads to reduced intracellular Dox levels, possibly due to the overexpression of energy-dependent drug efflux pump proteins such as P-gp [34]. The results exhibited that the covalent conjugation of Dox with [(WR)_8_WKβA] increased the cellular uptake and retention of Dox as compared with Dox alone (determined by co-localization of the blue signal of the DAPI-stained nuclei and the red signal of Dox). The physical mixture of peptide and free Dox showed less Dox localization compared to the conjugate in SK-OV-3 cells, as shown in Figure 2. Dox conjugate and the physical mixture showed localization mainly in the nucleus, the main site of action for Dox. The data suggest that [(WR)_8_WKβA]-Dox conjugate significantly enhanced the retention of Dox in the nucleus versus Dox. 

**Figure 3:**
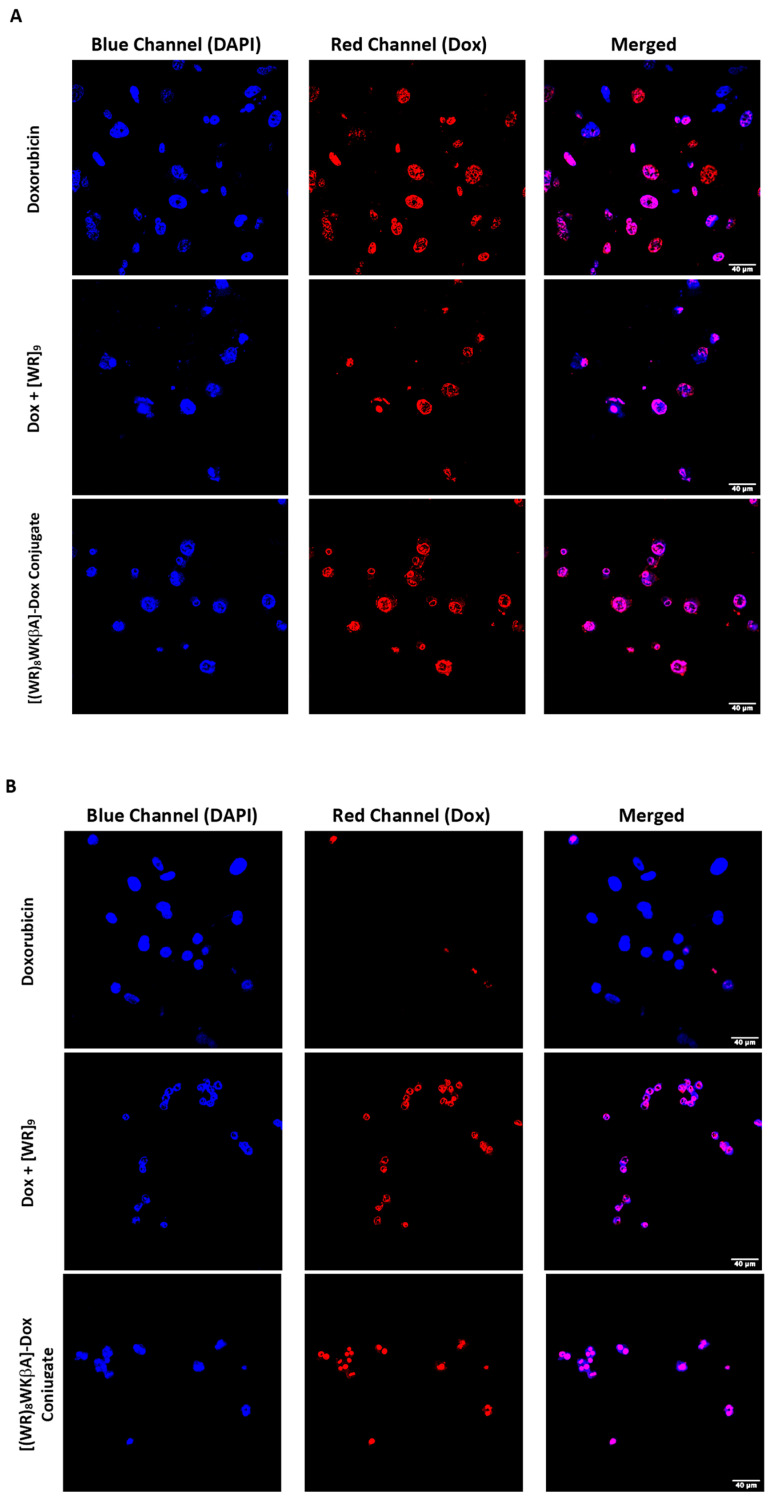
Confocal microscopy images of free Dox (5 μM), [WR]_9_ + Dox (1:1, (5 μM)), or [(WR)_8_WKβA]-Dox conjugate (5 μM) after 24 h in (**A**) MDA-MB-231 and (**B**) MES-SA/MX2 cells. Red represents the fluorescence of Dox.

We explored Dox internalization into Dox-resistant cell lines using confocal microscopy (Figure 3A,B). For this purpose, wild type MDA-MB-231 and MES-SA/MX2 cells were exposed to Dox (5 μM), [(WR)_8_WKβA]-Dox conjugate (5 μM), or [WR]_9_ + Dox (1:1, (5 μM)) for 24 h, and the nuclei were stained with DAPI. Indeed, the images demonstrated the internalization of free and conjugated Dox in wild type MDA-MB-231 cells, while only [(WR)_8_WKβA]-Dox conjugate and the physical mixture were internalized into the resistant cell lines overexpressing P-gp, which clearly indicates that this approach can potentially be used to overcome multidrug resistance mechanism in cancer cells, as shown in Figure 3A,B. These data are consistent with antiproliferative results described above, where [(WR)_8_WKβA]-Dox conjugate and [WR]_9_ + Dox exhibited significantly higher antiproliferative activity than Dox alone in Dox-resistant MES-SA/MX2 cells.

**Figure 4:**
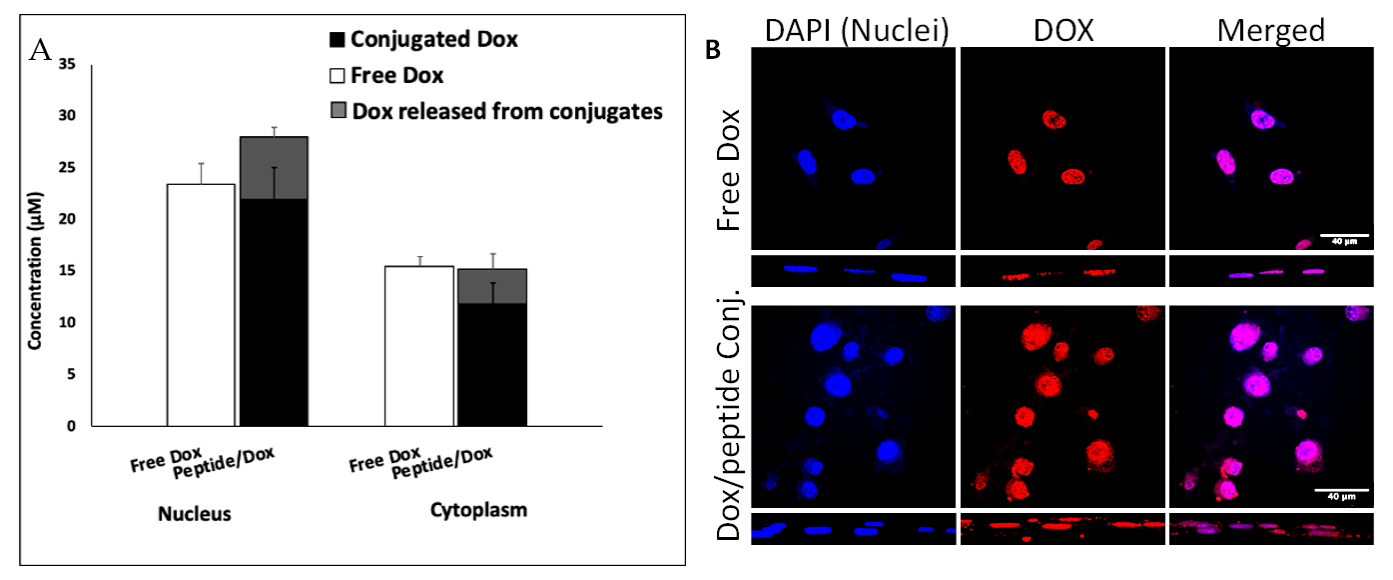
Intracellular tracking of Dox in MDA-MB-231 cells revealed efficient nuclear delivery by [(WR)_8_WKβA]-Dox via quantification by (**A**) HPLC in nuclear and cytoplasmic compartments and by (**B**) confocal microscopy.

We analyzed the localization of [(WR)_8_WKβA]-Dox (50 μM) in cellular compartments by isolation of nuclei and cytoplasm and using an HPLC method that differentially quantifies free and peptide-conjugated Dox (Figure 4A). While free Dox easily diffuses to the nucleus, the total concentration of Dox (conjugated and released) in the nucleus after 4 h exposure to [(WR)_8_WKβA]-Dox was higher than free Dox. The confocal microscopy images confirm the localization of peptide conjugated Dox in the nuclear compartment (Figure 4B).

#### 3.2.3. Mechanistic Studies of Cellular Internalization

Generally, cyclic cell-penetrating peptides (CPPs) have different uptake mechanisms depending on the physicochemical properties, the primary and secondary structure, concentration, membrane structure and type of cells, incubation time, and cargo type [35]. Two main mechanisms of permeation through cell membranes have been highly proposed in the literature: direct membrane translocation via energy-independent pathways, and endocytosis pathways, which require energy consumption [36]. Direct translocation occurs due to electrostatic interaction between positively charged residues of CPPs and negatively charged phospholipid bilayer, and is further classified into different models such as the carpet model, pore formation, and the inverted micelle model [37,38,39]. Endocytosis, on the other hand, is an energy-dependent and active mechanism composed of various pathways, including phagocytosis and pinocytosis, which are classified into macropinocytosis, clathrin-dependent endocytosis, caveolin-dependent endocytosis, and clathrin- and/or caveolin-independent endocytosis [40,41]. 

**Figure 5:**
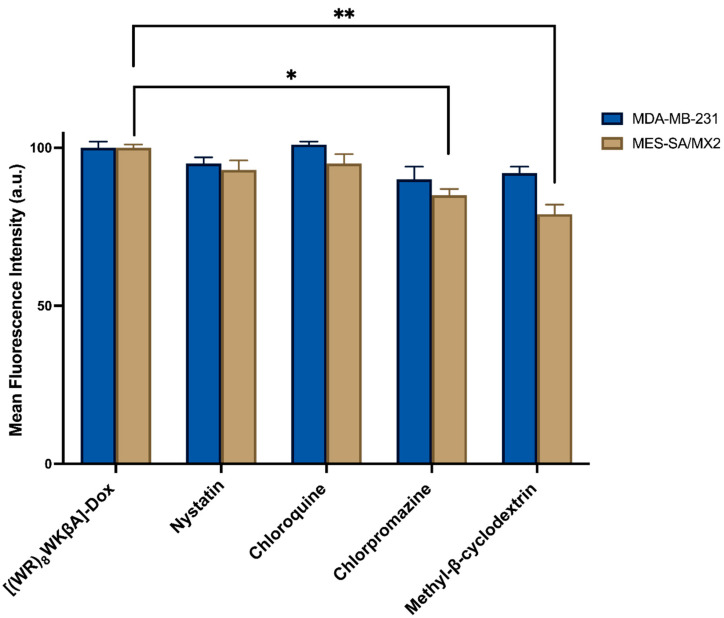
[(WR)_8_WKβA]-Dox conjugate cellular uptake in MDA-MB-231 and MES-SA/MX2 cells in the presence of different inhibitors of clathrin- and caveolae-dependent endocytosis, as studied by flow cytometry. Results are mean ± SD (*n* = 3) (* *p* < 0.05, ** *p* < 0.01, treatment vs. Ctrl ([(WR)_8_WKβA]-Dox conjugate alone).

Thus, flow cytometry and confocal microscopy studies were performed to further explore the mechanism of cellular internalization for [(WR)_8_WKβA]-Dox conjugate in Dox-sensitive/resistance cells. We studied the cellular internalization of [(WR)_8_WKβA]-Dox conjugate in MDA-MB-231 and MES-SA/MX2 cells in the presence of the following endocytosis inhibitors (Figure 5). Chlorpromazine is a well-known inhibitor of clathrin-mediated endocytosis; methyl-β-cyclodextrin acts as an inhibitor of caveolae-mediated uptake; chloroquine is an antimalaria medication that reduces the expression of phosphatidylinositol binding clathrin assembly protein, and is an inhibitor of endocytosis; and nystatin is a caveolae/lipid raft-dependent endocytosis inhibitor [42,43,44,45,46]. Cells were incubated with nystatin (50 μg/mL), chloroquine (100 μM), chlorpromazine (30 μM), and methyl-β-cyclodextrin (2.5 mM) for 30 min. The cells were then exposed to free Dox or peptide-Dox conjugate at 5 μM for 3 h.

The cellular uptake of [(WR)_8_WKβA]-Dox conjugate (5 µM) in the presence and absence of endocytosis inhibitors was not reduced significantly by nystatin and chloroquine, which inhibit caveolae-mediated endocytosis and clathrin-dependent endocytosis, respectively, in both MDA-MB-231 and Dox-resistant MES-SA/MX2 cell lines. However, a slight uptake reduction was observed in the presence of chlorpromazine and methyl-β-cyclodextrin in Dox sensitive MDA-MB-231 cells, and was statistically significant in Dox resistant MES-SA/MX2, as shown in the flow cytometry data (Figure 5). None of the endocytosis inhibitors were able to completely stop the cellular uptake of [(WR)_8_WKβA]-Dox conjugate. As a result, clathrin-mediated and caveolae-mediated endocytosis are excluded as the main mechanism of uptake. These data suggest direct penetration is the main mechanism involved in the internalization of [(WR)_8_WKβA]-Dox conjugate across the cell membrane, and that most of the uptake occurs through an endocytosis-independent manner.

**Figure 6:**
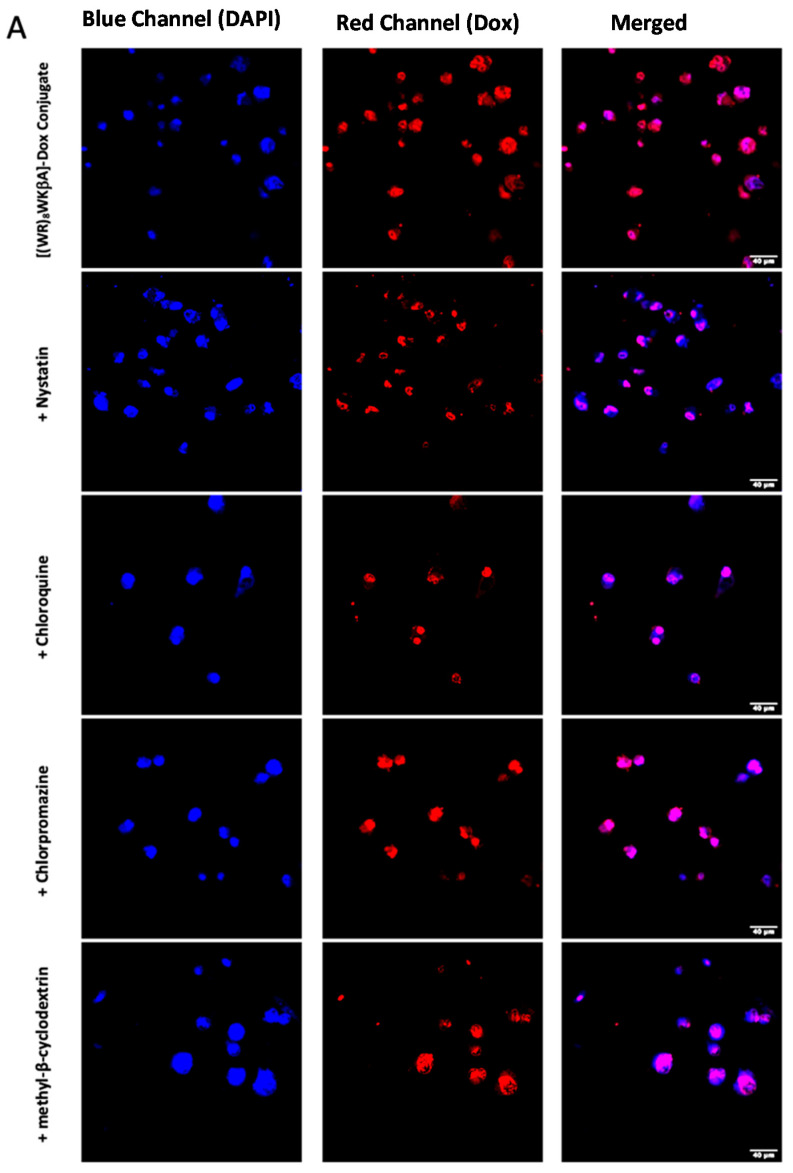

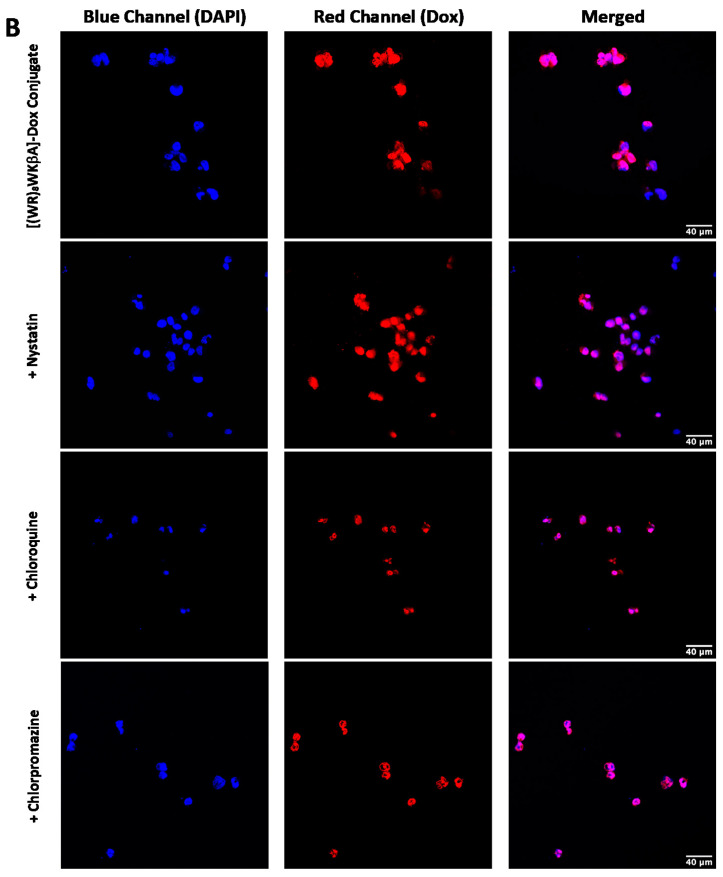
[(WR)_8_WKβA]-Dox conjugate (10 µM) cellular uptake in (**A**) MDA-MB-231 cells and (**B**) and MES-SA/MX2cells in the presence of different inhibitors of clathrin- and caveolae-dependent endocytosis, as studied by confocal microscopy.

We explored Dox internalization into MDA-MB-231 and MES-SA/MX2 cells in the presence and absence of endocytosis inhibitors using confocal microscopy. The confocal microscopy (Figure 6A,B) images demonstrate the localization of the conjugate in the nucleus, with no change in the uptake in the presence of chloroquine, chlorpromazine, nystatin, and methyl-β-cyclodextrin. These data are consistent with the flow cytometry findings.

#### 3.2.4. Drug Release and Plasma Stability of [(WR)_8_WKβA]-Dox Conjugate 

**Figure 7:**
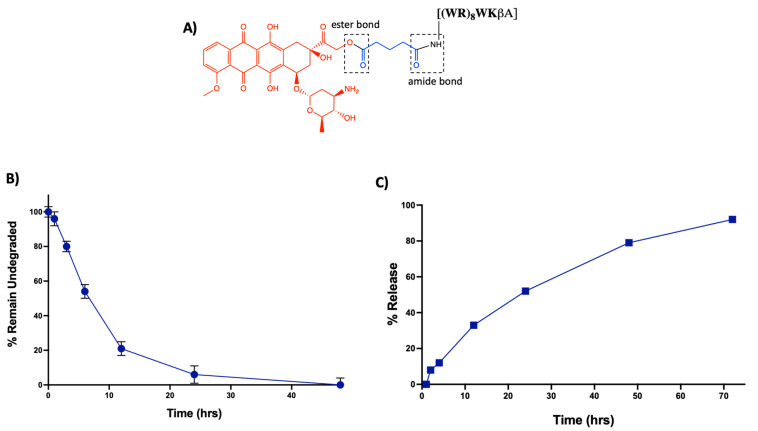
(**A**) Schematic representation of the possible susceptible bonds in [(WR)_8_WKβA]-Dox conjugate. (**B**) Stability analysis of [(WR)_8_WKβA]-Dox conjugate in human plasma. (**C**) Intracellular release of free Dox from [(WR)_8_WKβA]-Dox conjugate.

In addition to enhanced efficacy, the designing peptide-drug conjugate should be adequately stable to reach the target and release Dox intracellularly. The linker between the carrier peptide and cytotoxic drug is critically important. An ideal linker would maintain stability in the systemic circulation while allowing the drug to be released only after intracellular uptake of the conjugate by the cancer cells [47]. In [(WR)_8_WKβA]-Dox conjugate, an ester bond is utilized to attach Dox with a glutaryl linker, which is further attached to the peptide via an amide bond (Figure 7A). Ester and amide bonds are known to be susceptible to esterases and amidases, respectively, abundantly found in plasma and intracellular compartments such as the endosomes and lysosomes of cancer cells [48]. 

Thus, to determine the fate of the conjugate, we conducted a stability study mimicking the physiological environment using the human serum. Data are represented in the form of the percentage remaining of undegraded peptide-Dox conjugate against time by measuring area under the curve in analytical HPLC chromatogram (Figure 7B) (Figure S3, Supporting Information). [(WR)_8_WKβA]-Dox conjugate (5 µM) showed only 7% degradation after 1 h incubation with human plasma. However, 80% intact conjugate was observed at the 3 h time point. At 6 h, approximately 50% of the conjugate has been digested by enzymes present in human plasma. Half-life (t_1/2_) for [(WR)_8_WKβA]-Dox conjugate was (∼6 h), and only 6% of the conjugate was still intact at 24 h. Further studies are required to improve the stability of the conjugate. However, for this proof-of-concept study, the intact conjugate could remain stable enough to reach the target within 1–2 h.

In the same context, a cellular hydrolysis study was conducted to measure the release of free Dox in the cancer microenvironment. Intracellular hydrolysis for [(WR)_8_WKβA]-Dox conjugate was monitored in leukemia CCRF-CEM cells. The cells (1.37 × 10^7^) were first incubated with the conjugate (5 μM) for 4 h. Then, the incubation was continued with drug-free medium to determine the possibility of intracellular hydrolysis to free Dox. Data are represented in the form of percentage release of free Dox against time by measuring the area under the curve (AUC) in analytical HPLC chromatograms (Figure S4, Supporting Information). The cellular hydrolysis data exhibit that the [(WR)_8_WKβA]-Dox conjugate was hydrolyzed intracellularly and released Dox in a time-dependent manner. More than 36% of free Dox was released intracellularly within 12 h. Approximately 100% of Dox was released from the conjugate intracellularly within 72 h (Figure 7C). These data suggest that the uptake, retention, and sustained intracellular hydrolysis of [(WR)_8_WKβA]-Dox conjugate to free Dox contribute to the overall activity of the conjugate as a potential prodrug.

## 4. Conclusions

In this study, [(WR)_8_WKβA]-Dox conjugate was synthesized as a prodrug and evaluated against various cancer cell lines, including TNBC and Dox-resistant cells, in comparison to the free drug and the corresponding physical mixtures. The peptide–Dox conjugate demonstrated superior antiproliferative activity when compared with free Dox. The conjugate inhibited the cell proliferation of ovarian adenocarcinoma (SK-OV-3) by 59%, and the TNBC cells MB-MDA-231 and MCF-7 by 71% and 77%, respectively, at a concentration of 5 μM after 72 h of incubation. In contrast, Dox inhibited the proliferation of SK-OV-3, MDA-231, and MCF-7 by 35%, 63%, and 57%, respectively. Furthermore, [(WR)_8_WKβA]-Dox conjugate significantly reduced antiproliferative activity against Dox-resistant cells (MES-SA/MX2), by 92% when compared with free Dox, which decreased proliferation by only 15% at 5 μM. Confocal microscopy experiments and cellular uptake studies confirmed the antiproliferative results, demonstrating the internalization of the conjugate in TNBC and Dox-resistant cells. The cellular uptake was found to be mostly through an endocytosis-independent pathway. These data indicate that the conjugate has potential as a peptide-based prodrug in Dox-resistant cells

## Data Availability

Not applicable.

## Supplementary Materials

The following supporting information can be downloaded at: https://www.mdpi.com/article/10.3390/cells11020301/s1. Figure S1: Inhibition of heart cells (H9C2) by free Dox (5 µM), [(WR)_8_WKβA]-Dox, [WR]_9_ + Dox, and [WR]_9_ at 1, 5, and 10 μM. R; Figure S2: Confocal microscopy images of free Dox (5 μM), [WR]_9_ + Dox (1:1 and 1:2), or [(WR)_8_WKβA]-Dox conjugate (5 and 10 μM) after 24 h in H9C2 cell line; Figure S3: Stability of [(WR)_8_WKβA]-Dox conjugate at 5 μM in human plasma; Figure S4: Intracellular release of free Dox from [(WR)_8_WKβA]-Dox conjugate; Figure S5: Mass spectra of [(WR)8WKβA] peptide; Figure S6: Mass spectra of [(WR)_8_WKβA]-Dox conjugate; and Figure S7: Analytical HPLC purity profile of [(WR)_8_WKβA]-Dox conjugate.
