# Supplementary material for: [(WR)8WKβA]-Doxorubicin Conjugate: A Delivery System to Overcome Multi-Drug Resistance against Doxorubicin
Source: Cells. 2022 Jan 16;11(2):301. doi: 10.3390/cells11020301 (PMC8774489; doi:10.3390/cells11020301)
Supplement: Supplementary file 1 [file cells-11-00301-s001.zip › cells-1491796-supplementary.pdf]

SUPPORTING INFORMATION

[(WR)$_8$WKβA]-Doxorubicin Conjugate: A Delivery System to Overcome Multi-Drug Resistance against Doxorubicin

Khalid Zoghebi[1,2], Hamidreza Montazeri Aliabadi,[1] Rakesh Kumar Tiwari,[1,*] Keykavous Parang[1,*]

[1]Center for Targeted Drug Delivery, Department of Biomedical and Pharmaceutical Sciences, Chapman University School of Pharmacy, Harry and Diane Rinker Health Science Campus, Irvine, CA 92618, United States

[2]Department of Pharmaceutical Chemistry, College of Pharmacy, Jazan University, Jazan 82826, Saudi Arabia

*Corresponding Authors

Keykavous Parang, Pharm.D., Ph.D.
Chapman University School of Pharmacy
Harry and Diane Rinker Health Science Campus
#262, 9401 Jeronimo Road
Irvine, CA 92618, USA
Tel: (714) 516-5489. Fax: (714) 516-5481
E-mail: parang@chapman.edu

Rakesh K. Tiwari, Ph.D.
Chapman University School of Pharmacy
Harry and Diane Rinker Health Science Campus
#263, 9401 Jeronimo Road
Irvine, CA, 92618, U.S.A.
Tel: (714) 516-5483. Fax: (714) 516-5481
E-mail: tiwari@chapman.edu

Table of Contents

Page

Cytotoxicity assay in heart cells (H9C2) ……………………..……………………….………….S3

Cellular internalization of [(WR)$_8$WKβA]-Dox conjugate using Confocal microscopy in heart cells (H9C2) ………………………………………………………..………………………S4

Stability Results ………………………………………………………………………………S5

Hydrolysis Results ……………………………………………………………………………S6

Mass spectra for [(WR)$_8$WKβA] peptide ……………………………………………………..S7

Mass spectra for [(WR)$_8$WKβA]-Dox conjugate …………………………………………..S8

HPLC purity profile of [(WR)$_8$WKβA]-Dox conjugate ……………………………………S9

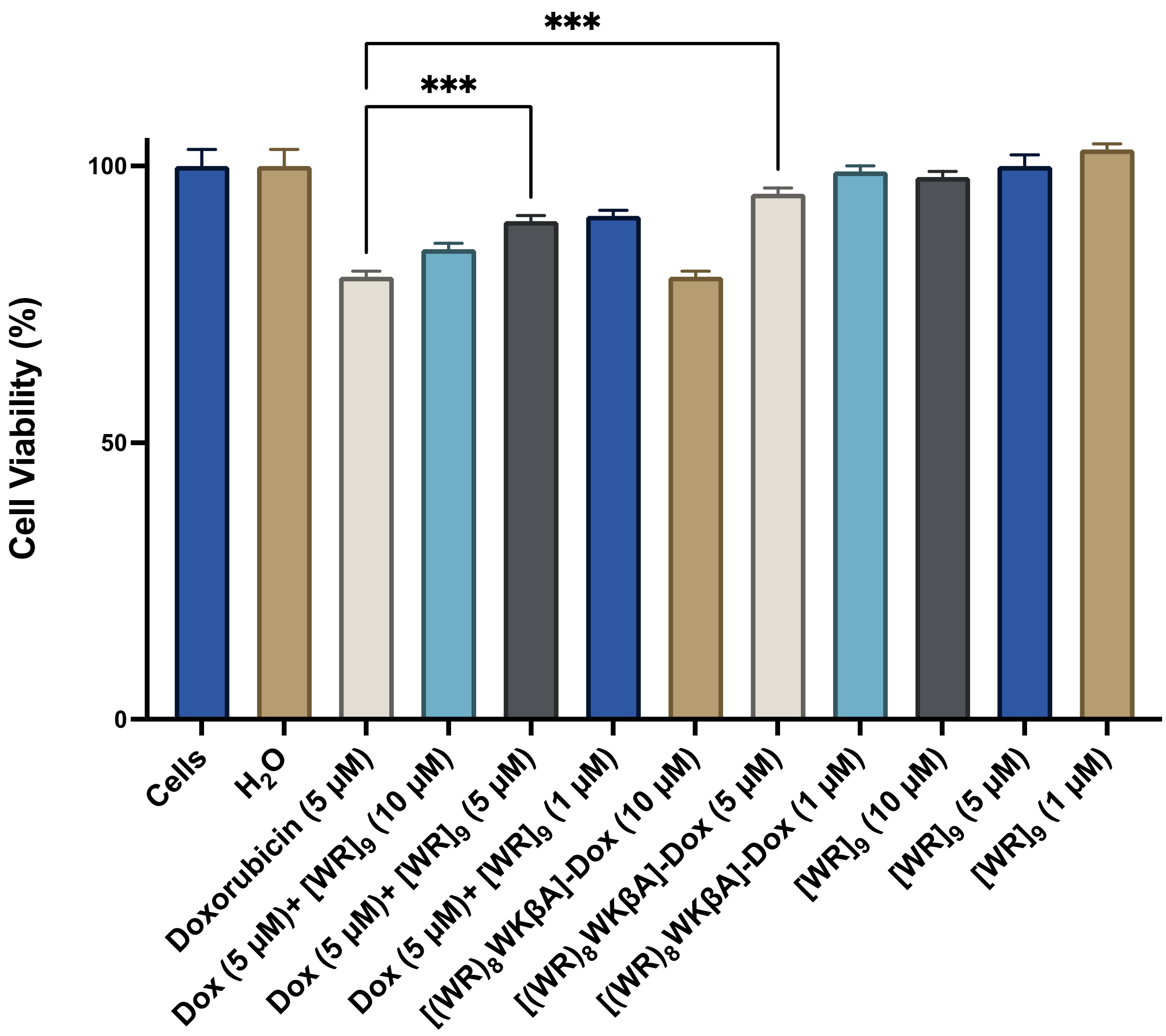

Figure S1. Inhibition of heart cells (H9C2) by free Dox (5 µM), $[(WR)_8WK\beta A]$-Dox, $[WR]_9$ + Dox, and $[WR]_9$ at 1, 5, and 10 µM. Results are mean ± SD (n = 3). (***p<0.001 treatments at 5 µM vs. Ctrl (Dox) at 5 µM).

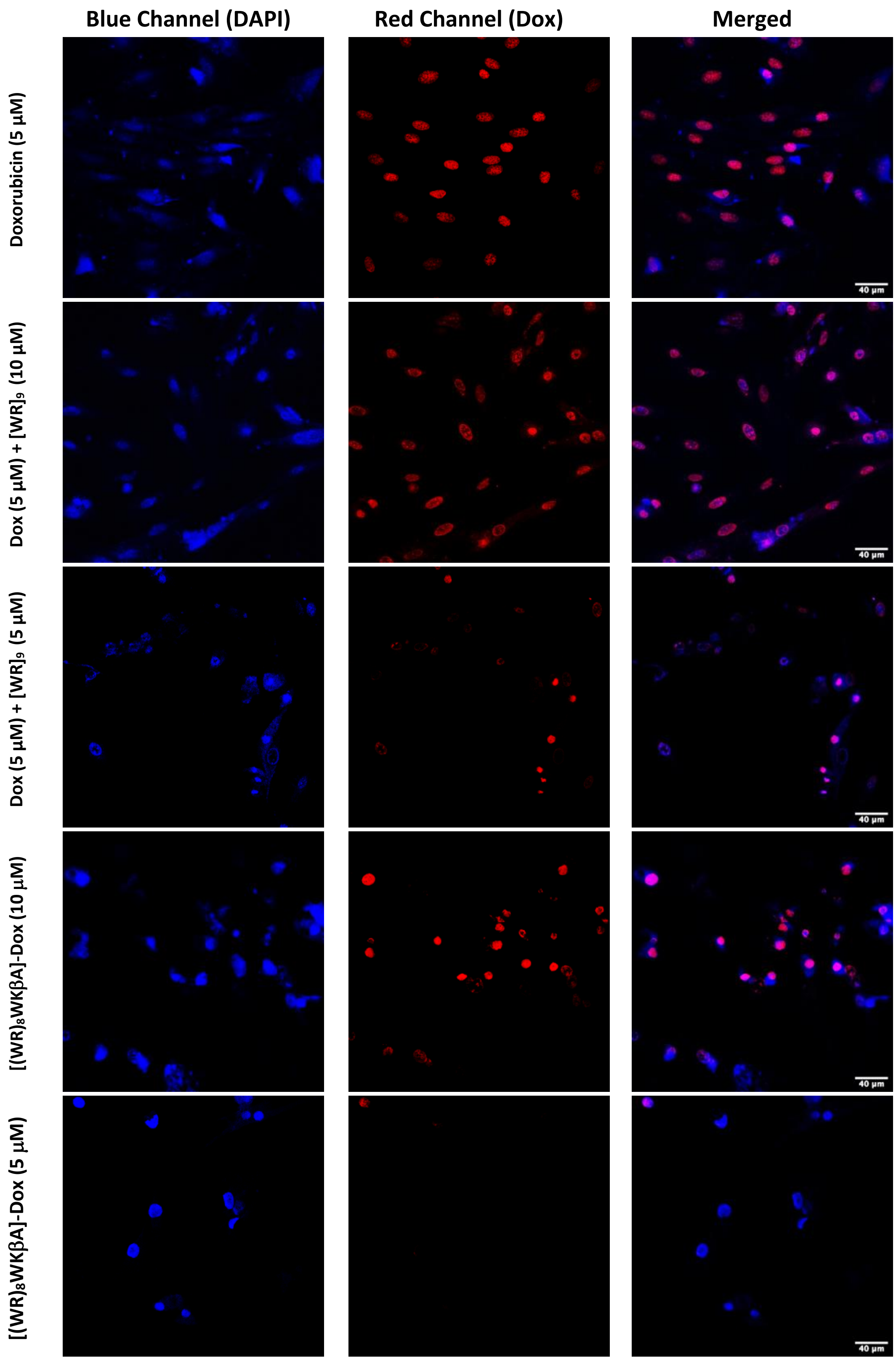

Figure S2. Confocal microscopy images of free Dox (5 μM), $[WR]_9$ + Dox (1:1 and 1:2), or $[(WR)_8WK\beta A]$-Dox conjugate (5 and 10 μM) after 24 h in H9C2 cell line.

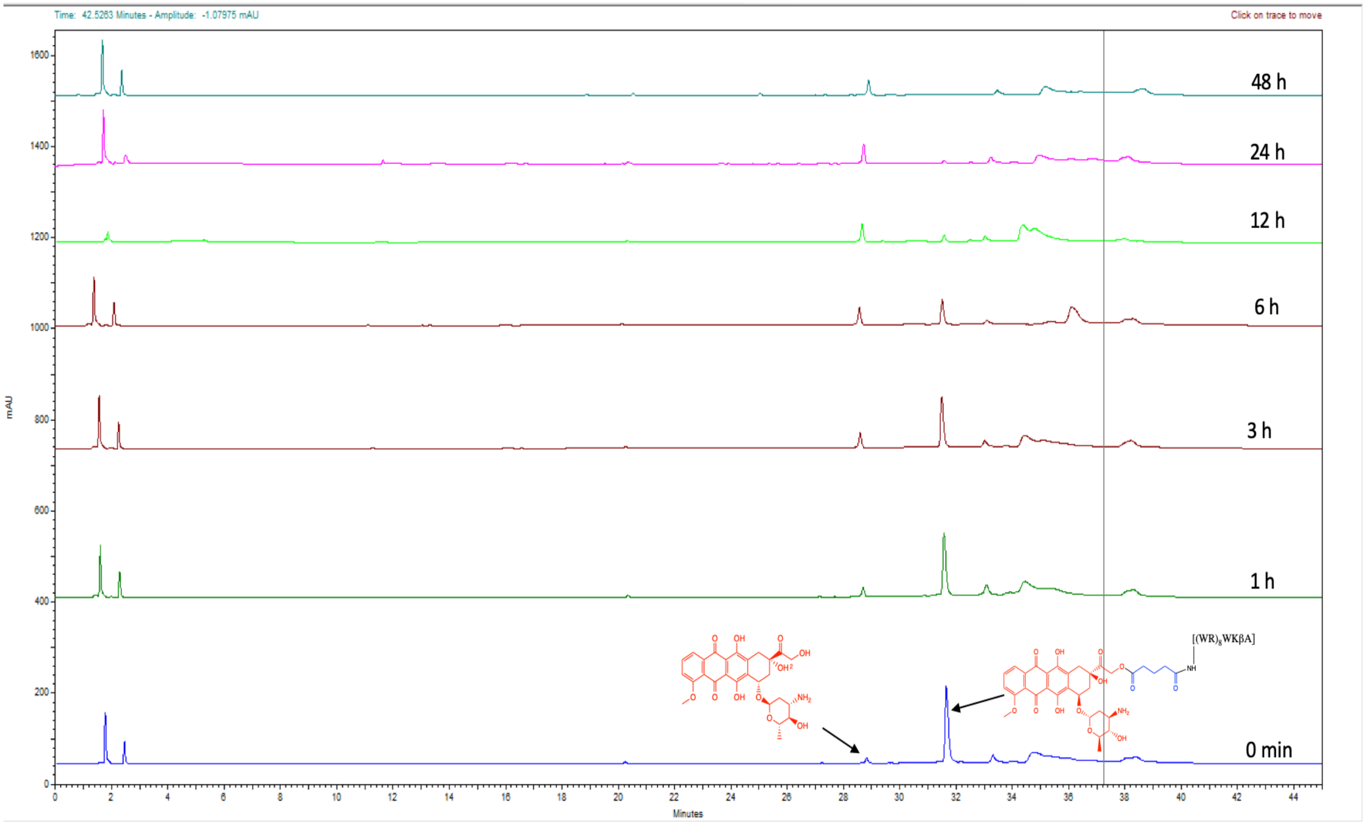

Figure S3. Stability of [(WR)$_8$WKβA]-Dox conjugate at 5 μM in human plasma.

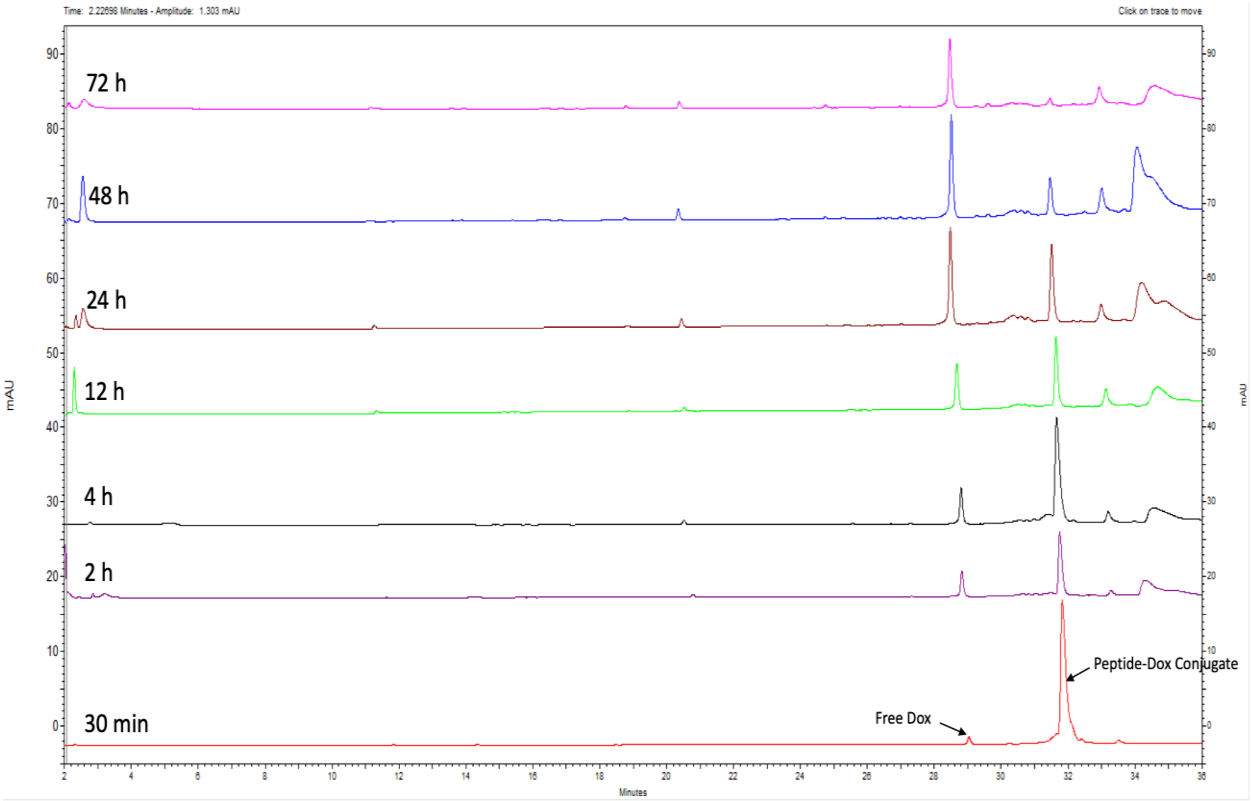

Figure S4. Intracellular release of free Dox from [(WR)$_8$WKβA]-Dox conjugate.

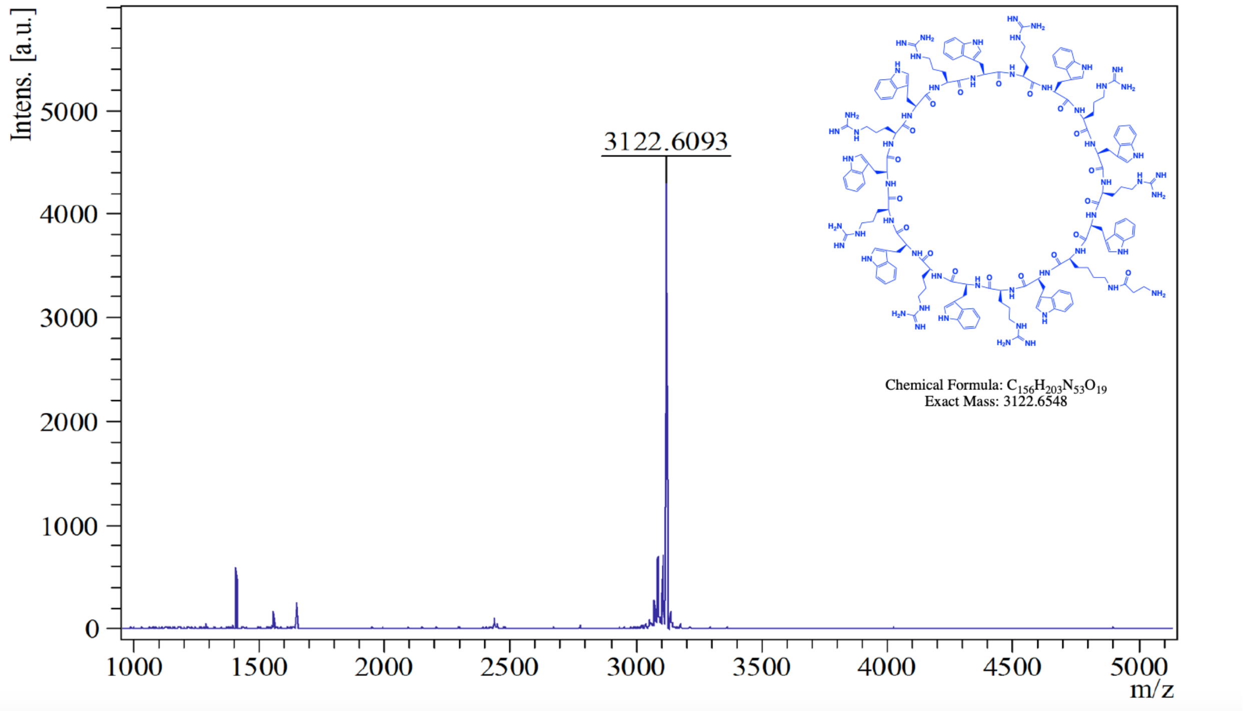

Figure S5. Mass spectra of [(WR)$_8$WKβA] peptide.

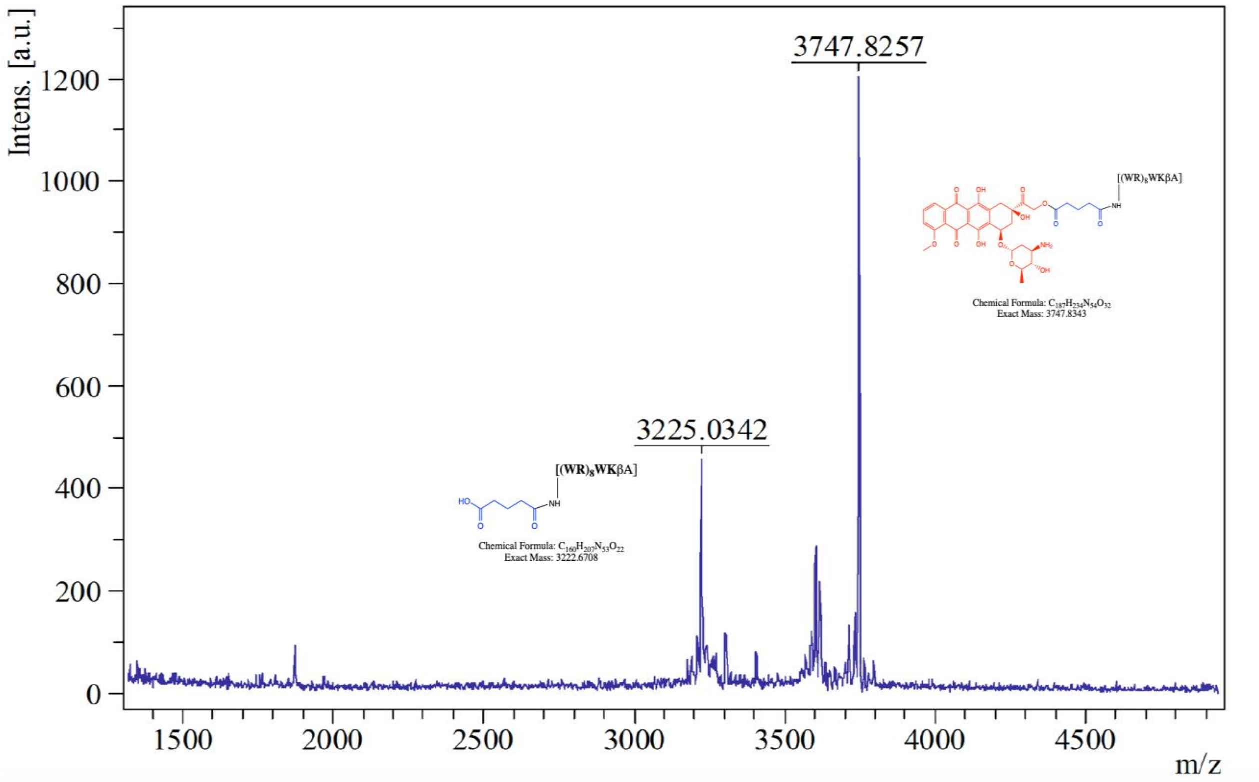

Figure S6. Mass spectra of [(WR)$_8$WKβA]-Dox conjugate.

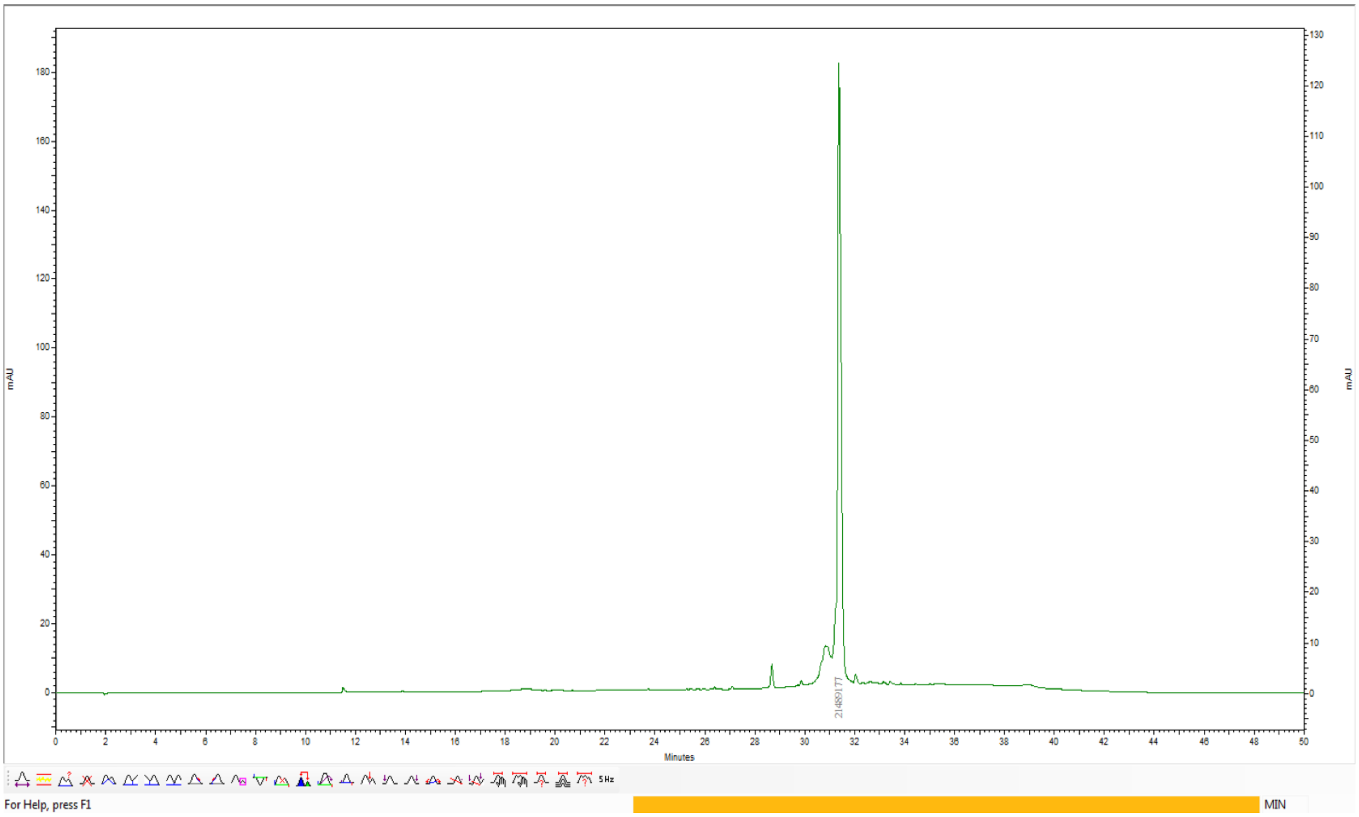

Figure S7. Analytical HPLC purity profile of [(WR)$_8$WKβA]-Dox conjugate.
